# MiR-193b promoter methylation accurately detects prostate cancer in urine sediments and miR-34b/c or miR-129-2 promoter methylation define subsets of clinically aggressive tumors

**DOI:** 10.1186/s12943-017-0604-0

**Published:** 2017-01-31

**Authors:** Jorge Torres-Ferreira, João Ramalho-Carvalho, Antonio Gomez, Francisco Duarte Menezes, Rui Freitas, Jorge Oliveira, Luís Antunes, Maria José Bento, Manel Esteller, Rui Henrique, Carmen Jerónimo

**Affiliations:** 1 Cancer Biology and Epigenetics Group, IPO Porto Research Center (CI-IPOP), Portuguese Oncology Institute of Porto (IPO Porto), Research Center-LAB 3, F Bdg., 1st floor, Rua Dr António Bernardino de Almeida, 4200-072 Porto, Portugal; 2 Cancer Epigenetics and Biology Program, Bellvitge Biomedical Research Institute, Barcelona, Catalonia Spain; 3 Biomedical Sciences Graduate Program, Institute of Biomedical Sciences Abel Salazar– University of Porto (ICBAS-UP), Porto, Portugal; 4 Departments of Pathology, Portuguese Oncology Institute of Porto (IPO Porto), Porto, Portugal; 5 Departments of Urology, Portuguese Oncology Institute of Porto (IPO Porto), Porto, Portugal; 6 Departments of Epidemiology, Portuguese Oncology Institute of Porto (IPO Porto), Porto, Portugal; 7 Department of Pathology and Molecular Immunology, Institute of Biomedical Sciences Abel Salazar– University of Porto (ICBAS-UP), Porto, Portugal

**Keywords:** Biomarkers, Aberrant miR’s promoter methylation, Detection, Prognosis, Prostate cancer

## Abstract

**Background:**

Contemporary challenges of prostate cancer (PCa) include overdiagnosis and overtreatment, entailing the need for novel clinical tools to improve risk stratification and therapy selection. PCa diagnosis and prognostication might be perfected using epigenetic biomarkers, among which aberrant DNA methylation of microRNA promoters has not been systematically explored. Herein, we identified aberrantly methylated microRNAs promoters in PCa and assessed its diagnostic and prognostic biomarker potential.

**Methods:**

Using HumanMethylation450 BeadChip-based analysis differentially methylated CpGs in microRNA promoters were identified. Promoter methylation of six microRNAs (miR-34b/c, miR-129-2, miR-152, miR-193b, miR-663a and miR-1258) was analyzed by qMSP in three sets (180 prostatectomies, 95 urine sediments and 74 prostate biopsies). Biomarkers’ diagnostic (validity estimates) and prognostic [disease-free (DFS) and disease-specific survival (DSS)] performance was assessed.

**Results:**

Significantly higher promoter methylation levels in PCa were confirmed for six candidate microRNAs. Except for miR-152, all displayed AUC values higher than 0.90, with miR-1258 and miR-193b disclosing the best performance (AUC = 0.99 and AUC = 0.96, respectively). In urine samples, miR-193b showed the best performance (91.6% sensitivity, 95.7% specificity, AUC = 0.96). Moreover, higher miR-129-2 independently predicted for shorter DSS and miR−34b/c methylation levels independently predicted for shorter DFS and DSS.

**Conclusions:**

Quantitative miR-193b, miR-129-2 and miR-34b/c promoter methylation might be clinically useful PCa biomarkers for non-invasive detection/diagnosis and prognostication, both in tissue and urine samples.

**Electronic supplementary material:**

The online version of this article (doi:10.1186/s12943-017-0604-0) contains supplementary material, which is available to authorized users.

## Background

Prostate cancer (PCa) is the most incident male cancer in western countries, constituting the second most common cause of cancer and the sixth leading cause of death by cancer among men worldwide [1]. For 2012, it was estimated that PCa alone accounted for 420,000 newly diagnosed cancer cases and 101,000 of all cancer-related deaths in European men [2]. PCa is age-related and very heterogeneous, both molecularly and clinically, ranging from relatively indolent to highly aggressive. It is typically asymptomatic at its earliest stages, when adequate treatment is mostly curative, in contrast with its late diagnosis, which usually impairs a curative-intent therapeutic strategy [3]. This led to the widespread use of serum PSA as screening tool for PCa. However, it is now commonly accepted that this entailed overdiagnosis and overtreatment, justifying the strong recommendation against PCa screening and prompting the search for more effective biomarkers [4].

DNA methylation is a chemically stable and easily quantified alteration [5]. We and others have previously reported on the use of quantitative promoter methylation of several protein-coding genes for early diagnosis and prognostication of PCa [6]. Although several gene methylation panels have been then developed [7, 8], both sensitivity and specificity must be perfected to allow for clinical translation.

MicroRNAs, a class of small (19–25 nucleotides) non-coding RNA, are involved in virtually all cellular processes and frequently deregulated in cancer cells [9], although its abrogation due to aberrant promoter methylation has been seldom reported [10]. Because this epigenetic alteration is likely to be highly cancer-specific, it might constitute an effective cancer biomarker. Thus, we aimed to explore the potential of microRNA-coding genes promoter methylation as diagnostic and prognostic biomarkers in PCa. Therefore, after genome-wide screening, a set of putative tumor-suppressor microRNAs (miR-34b/c, miR-129-2, miR-152, miR-193b, miR-663a and miR-1258) with increased promoter methylation levels in PCa compared to normal prostate tissues was identified and further validated in clinical samples.

## Methods

### Patients and samples collection

For the purposes of this study, three independent cohorts of PCa patients were defined.

PCa tissue samples were prospectively collected from 180 patients with clinically localized disease, consecutively diagnosed and submitted to radical prostatectomy (RP) from 2001 to 2006, at Portuguese Oncology Institute of Porto (Cohort #1). Fifteen control samples were obtained from cystoprostatectomy specimens with bladder cancer, not harbouring PCa nor prostatic involvement by urothelial carcinoma (morphologically normal prostate tissue, MNPT). After collection, tissue samples were fresh-frozen at −80 °C and subsequently cut in a cryostat for DNA extraction. Prostate biopsy samples were collected from 74 PCa suspects (elevated serum PSA), referred to Portuguese Oncology Institute - Porto from 2001 to 2003 (Cohort #2). In addition to standard diagnostic cores, a core was collected from the most suspicious area, fresh-frozen at −80 °C and subsequently cut in a cryostat for DNA extraction.

Voided urine samples from 95 PCa patients were collected from 1999 to 2002 (Cohort #3). The control set is composed of urine samples collected from 17 healthy donors and 29 patients without urological malignancy. Samples were centrifuged at 4,000 rpm for 20 min, washed in PBS 1× and the pellets were frozen at −80 °C.

Clinical data was retrieved from clinical charts. Survival data was collected for patients of Cohort #1 and of Cohort #2. Disease-specific survival (DSS) time was calculated as the time elapsed since diagnosis until death or the last follow-up. Disease-free survival (DFS) was calculated from the date of the radical prostatectomy or other curative treatment to the date of biochemical relapse, date of last follow-up, or death if relapse-free.

All patients enrolled (Tables 1 and 2) signed informed consent. This study was approved by institutional review board (CES-IPOPFG-EPE 019/08 and CES-IPOPFG-EPE 205/2013).

**Table 1:** Clinical and pathological data of tissue and urine samples used in this study

	Prostatectomies		Urine samples	
Clinicopathological data	MNPT	PCa^a^	Controls	PCa^b^
Patients, *n*	15	180	46	95
Median age, *years* (range)	63 (45–80)	65 (49–74)	61 (58–77)	64 (45–80)
Median PSA (*ng/mL*) (range)	-	8.3 (3.4-23.0)	-	8.8 (3.5-20.4)
Pathological Stage				
pT2 (%)	-	96 (53.3)	-	46 (48.4)
pT3 (%)	-	84 (46.7)	-	49 (51.6)
Gleason score				
<7 (%)	-	56 (31.1)	-	37 (39.0)
≥7 (%)	-	124 (68.9)	-	58 (61.0)

^a^Cohort #1; ^b^Cohort #3

**Table 2:** Clinical and pathological data of Cohort #2 (prostate biopsies)

Patients, n	74
Median age, years (range)	68 (49–85)
Median PSA (ng/mL) (range)	18.22 (4.52-542)
Clinical stage	
T2 (%)	48 (64.9)
T3/T4 (%)	26 (35.1)
Gleason score	
<7 (%)	30 (40.5)
≥7 (%)	44 (59.5)
Follow up	
Median (months) (range)	114.9 (10.3–170.1)
Patients without remission (%)	3 (4)
Biochemical recurrence (%)	29 (39.2)
Death due to PCa (%)	13 (17.6)
Therapy	
Surgery (%)	17 (23.0)
ADT (%)	35 (47.3)
Radiotherapy (%)	4 (5.4)
ADT + Radiotherapy (%)	17 (23.0)
Radiotherapy + Criotherapy (%)	1 (1.3)
CAPRA Score	
Low-risk (0–2)	7 (9.5)
Intermediate-risk (3–5)	26 (35.1)
High-risk (6–10)	41 (55.4)

ADT- androgen deprivation therapy

### Nucleic acid isolation, bisulphite treatment, HumanMethylation 450 BeadChip and qMSP analysis

DNA extracted by phenol-chloroform as described elsewhere[11] was chemically modified using sodium bisulfite with EZ DNA Methylation-Gold™ Kit (Zymo Research, CA, USA) according to manufacturer’s protocol.

HumanMethylation450 BeadChip (Illumina, USA) allowed for gene methylation profiling of tissue samples (5 controls and 25 tumors), using 500 ng of bisulphite-converted DNA, according to manufacturer’s instructions. DNA methylation levels were depicted as beta-values ranging from 0–1.

Validation of all candidates was performed by quantitative methylation using KAPA SYBR FAST qPCR Kit (Kapa Biosystems, MA, USA). All reactions were run in triplicates in 384-well plates using Roche LightCycler 480 II, with *β-actin* (*ACTB*) as internal reference gene for normalization. Primer sequences (Additional file 1: Table S1) were designed using Methyl Primer Express 1.0 and purchased from Sigma-Aldrich (MO, USA).

### Statistical analysis

For HumanMethylation 450 BeadChip data, a threshold intensity with *P*-value ≤ 0.01 was considered for further analysis. To identify consistently differentially methylated CpG sites, Wilcoxon rank sum paired test was performed for normalized beta-values. *P*-values were adjusted using false discovery rate, and CpGs with *P*-values <0.05 were selected.

In Cohort #1, pathological variables were categorized [Gleason score (GS): <7 and ≥7; pathological stage: pT2 and pT3]. Kruskall-Wallis and Mann–Whitney U tests allowed for comparisons among three or more groups and between two groups, respectively. For multiple comparisons P values were adjusted according to Bonferroni’s correction. Spearman nonparametric correlation was performed to ascertain association between methylation and PSA serum levels.

In Cohort #1 and Cohort #3, receiver operator characteristics (ROC) curves were constructed by plotting true positive rate (sensitivity) against false positive rate (1-specificity) and area under the curve (AUC) was calculated to assess diagnostic performance. Biomarker validity estimates [specificity, sensitivity, positive predictive value, negative predictive value and accuracy] were determined using as cut-off the highest value obtained through ROC curve analysis [sensitivity + (1-specificity)].

In Cohort #1 and Cohort #2, DSS and DFS curves were built using Kaplan–Meier method and the prognostic significance of clinicopathological variables (clinical stage, GS and serum PSA in both cohorts, and CAPRA Score in Cohort #2) was assessed using log-rank test. CAPRA score values were categorized as 0–2 (low-risk), 3–5 (intermediate risk) and 6–10 (high-risk) [12]. To test the prognostic significance of miR-34b/c and miR-129-2 promoter methylation, samples were categorized based on methylation levels of each miR (using percentile 75 as threshold) [11]. A Cox-regression model comprising all variables (multivariable analysis) was constructed. SPSS Statistics 20 (IBM, NY, USA) was used for all statistical analyses and graphics were assembled using GraphPad 5 Prism (GraphPad Software, CA, USA). *P* values <0.05 were considered statistically significant.

## Results

### MicroRNA promoter hypermethylation in Radical Prostatectomy samples (Cohort #1)

**Fig. 1:**
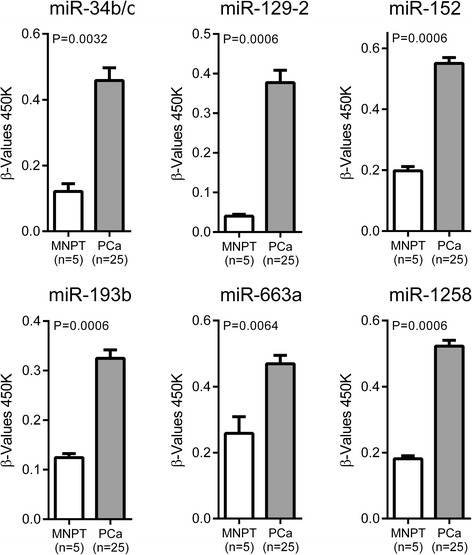
HumanMethylation450 BeadChip results. The microRNAs that displayed the most significant differences between normal and neoplastic samples were selected for further analysis. All data are presented as mean + standard deviation of the samples analyzed in each group

**Fig. 2:**
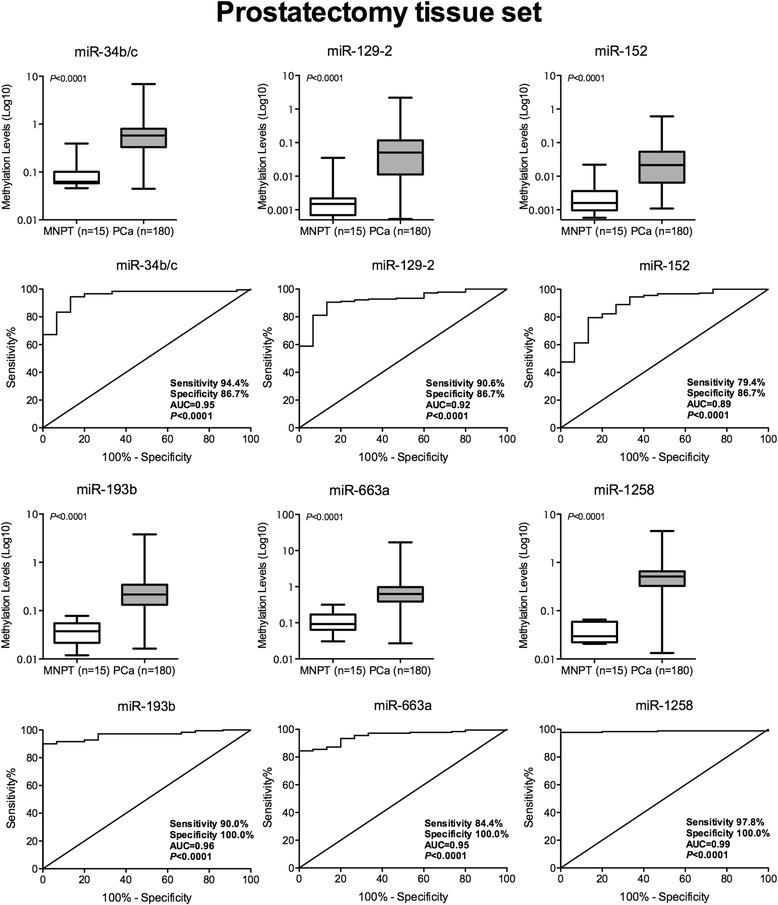
Box-plots and ROC curves for miR-34b/c, miR-129-2, miR-152, miR-193b, miR-663a and miR-1258 promoter methylation levels in morphologically normal prostate tissue (MNPT) and malignant (PCa) prostatic tissues from Cohort #1

Using the 450 K array, we screened microRNA *loci* regulated by DNA methylation in PCa. The microarray dataset included 5 MNPT and 25 PCa tissue samples. Candidate miRNAs were selected according to adjusted *P*-values and differences in the methylated fraction between MNPT and PCa tissues. CpG sites displaying statistically significant differences with adjusted *P*-values and mean methylation <0.3 in MNPT were further considered relevant. Among these, methylation sites located in the promoter region and in proximity to transcription start sites (TSS) [1500 and 200 base pairs upstream of TSS (TSS200; TSS1500 region)] were identified. For the validation study, we selected microRNAs in which significant differences (*P* < 0.05) in methylation levels were observed at all CpG sites mapped and differences in methylated fractions were >0.12. Thus, six microRNAs - miR-34b/c, miR-129-2, miR-152, miR-193b, miR-663a, and miR-1258 (Fig. 1; Table 3), were selected for large scale validation in 15 MNPT and 180 PCa samples (Cohort #1). In this series, overall methylation levels remained significantly increased in PCa compared with MNPT (Fig. 2), confirming the results of the array analysis.

**Table 3:** MicroRNA/CpG island probe distribution derived from the Infinium HumanMethylation450 BeadChip

miR	TargetID	Probe Sequence	Chr	UCSC Refgene Name	UCSC Refgene Accession	UCSC Refgene Group	UCSC CpG Islands Name	Relation to UCSC CpG Island
miR-34b/c	cg22879515	GTCCTCCCCGGCAGCGCCGCCCGCTGGCCCAGCTACGCGTGTTGTGCGCTGCGAGGCCGG[CG]GGGGTCCCGCTGGGCCCGGGGGTGTCCTCGGGGGCCGCTTGCGCCCAGCCATGGTAGGGC	11	MIR34B; BTG4; MIR34C	NR_029839; NM_017589;NR_029840	TSS200; TSS1500; TSS1500	chr11:111383168–111383892	Island
miR-129-2	cg14416371	GAGACACGAGTCCAGGGGCGCGGAGGGGCGGGCAGCGCGCGGAGTGGTGAGACTGAGCCG[CG]ATGGAACGCGCTGGGGAGACCCAGCCTGTTCGGCTCCAGGGTTCGGAGACATCCTGGGCT	11	MIR129-2	NR_029697	TSS200	chr11:43602545–43603215	Island
miR-152	cg05687686	CAGCTTCGGCATATTTGGCGGAGCCGGGAAGGCCCGGAGCGCAAGAAGGCATCGCAGCCT[CG]CAGCAGATCTGAAAGGGTTGTGGGCGGGGGGCTCATTTTCGCCGGATTTCTTTTCCGTGT	2	MIR1258; ZNF385B	NR_031659;NM_152520	TSS1500; TSS1500	chr2:180725717–180726465	S_Shore
miR-193b	cg09918657	AGTGGCGTTTCTGGTTTCTCTTTGCTTCCAATCCCCACCAAGCGGAGCGTTGGAATGCGC[CG]CTTATGTCCTCTGAGGACACATCCATATTTATAATTTATTTTTAGGAGAAGTTGTGAAAA	16	MIR193B	NR_030177	TSS1500	chr16:14395604–14397075	S_Shore
miR-663a	cg08304190	GCCTCACGAGCCCCTGGTCCCGCCACCGGGGCCCCGAAGCGACCACAGCCACAAACTCAA[CG]CCAGGGCCACATCGCTCGTGATTCTCGTCCATCCTCCGACCCGGTCCCGCTCCGGGAGAC	20	MIR663	NR_030386	TSS200	chr20:26188638–26190348	Island
miR-1258	cg05850656	ACTGCTCCAGAGCCCGAGTCGGAGTGTATCACAGAACCTGGGCCGGGGGGGACAGCGGGC[CG]AGCCTCCTTCTTCCAGCTGATCCCTGGCCGGGCTGGACCTGCGCTATCAGCGCGCCCCCA	17	MIR152; COPZ2	NR_029687;NM_016429	TSS200; Body	chr17:46114573–46115059	Island

To assess the diagnostic potential of microRNA promoter methylation in PCa we performed ROC curve analysis (Fig. 2 and Table 4), which revealed AUC values ranging from 0.89 to 0.99, with miR-1258 (AUC = 0.99), miR-193b (AUC = 0.96) and miR-34b/c (AUC = 0.95) demonstrating the best performance. Because AUC for miR-152 was lower than 0.90, it was excluded from further analyses. Concerning validity estimates, miR-1258 promoter methylation levels displayed the highest values (97.8% sensitivity, 100% specificity) for PCa detection. Panels composed by two or more microRNAs did not improve performance (data not shown).

**Table 4:** Validity estimates for miR’s promoter methylation as markers for PCa in Cohort #1

microRNA	Sensitivity % (n positive/n total)	Specificity (%)	PPV (%)	NPV (%)	Accuracy (%)
miR-34b/c	94.4 (170/180)	86.7	98.8	56.5	93.8
miR-129-2	90.6 (163/180)	86.7	98.8	43.3	90.3
miR-152	79.4 (143/180)	86.7	98.6	26.0	80.0
miR-193b	90.0 (162/180)	100.0	100.0	45.5	90.8
miR-663a	84.4 (152/180)	100.0	100.0	34.9	85.6
miR-1258	97.8 (176/180)	100.0	100.0	78.9	97.9

*PCa* Prostate cancer; *PPV* positive predictive value; *NPV* negative predictive value

**Fig. 3:**
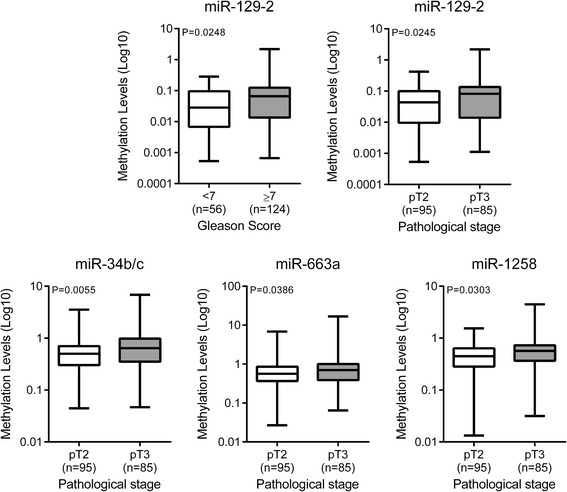
Distribution of methylation levels of microRNAs according to Gleason score and pathological stage in the series of patients submitted to radical prostatectomy (Cohort #1)

Then, we evaluated whether microRNA promoter methylation levels were associated with clinicopathological parameters. MiR-129-2 promoter methylation was associated with higher GS and pathological stage (*P* = 0.0248 and *P* = 0.0245, respectively), whereas, miR-34b/c, miR-663a and miR-1258 promoter methylation levels were only associated with higher pathological stage (*P* = 0.0055, *P* = 0.0386 and *P* = 0.0303, respectively) (Fig. 3).

To determine whether microRNA promoter methylation was PCa-specific, malignant and benign tissue samples from bladder (43 and 7) and kidney (50 and 9) were analyzed. MiR-34b/c, miR-193b and miR-1258 promoter methylation levels were significantly higher in PCa tissues compared to all other samples tested. Interestingly, miR-129-2 and miR-663a showed higher methylation levels in bladder cancer and were, thus, considered unsuitable for accurate detection of PCa in urine sediments (Additional file 2: Figure S1).

### MicroRNA promoter methylation in urine sediments (Cohort #3)

**Fig. 4:**
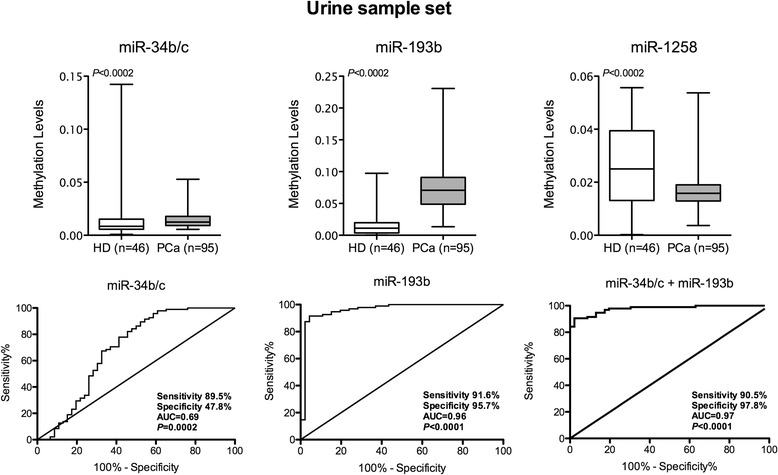
Box-plots and ROC curves of miR-34b/c, miR-193b and miR-1258 promoter methylation levels across urine sediments of controls (HD) and prostate cancer patients (PCa) from Cohort #3. Receiver operator characteristic (ROC) curves were constructed to evaluate the performance of the gene promoter methylation panel (miR-34b/c+miR-193b)

Best performing PCa-specific microRNAs - miR-34b/c, miR-193b and miR-1258 – were then tested in urine sediments, collected without previous prostatic massage, from PCa patients (*n* = 95, Cohort #3) and controls (*n* = 46). Higher miR-34b/c and miR-193b methylation levels and lower miR-1258 promoter methylation levels were depicted in PCa patients (Fig. 4). MiR-193b promoter methylation displayed the best performance with high sensitivity (91.6%) and specificity (95.7%), providing an overall accuracy of 92.9% (AUC = 0.96). Moreover, the panel including both miRs (miR-34b/c, miR-193b) augmented specificity (97.8%) and positive predictive value (98.9%) (Table 5). Addition of miR-34b/c promoter methylation did not improve biomarker performance. No associations between microRNAs’ promoter methylation levels and clinicopathological parameters were depicted in this series.

**Table 5:** Validity estimates for miR’s promoter methylation as markers for PCa in urine samples (Cohort #3)

microRNA	Sensitivity % (n positive/n total)	Specificity (%)	PPV (%)	NPV (%)	Accuracy (%)
miR-34b/c	89.5 (85/95)	47.8	78.0	68.8	75.9
miR-193b	91.6 (87/95)	95.7	97.8	84.6	92.9
miR-34b/c+miR-193b	90. 5% (86/95)	97.8	98.9	83.3	92.9

### MicroRNA promoter methylation as prognostic biomarker (Cohorts # 1 & # 2)

Owing to its association with stage and GS, the prognostic value of miR-34b/c and miR-129-2 promoter methylation was further tested in the set of 180 radical prostatectomy (Cohort #1) and in a prospective group of 74 PCa suspects (Cohort #2).

**Fig. 5:**
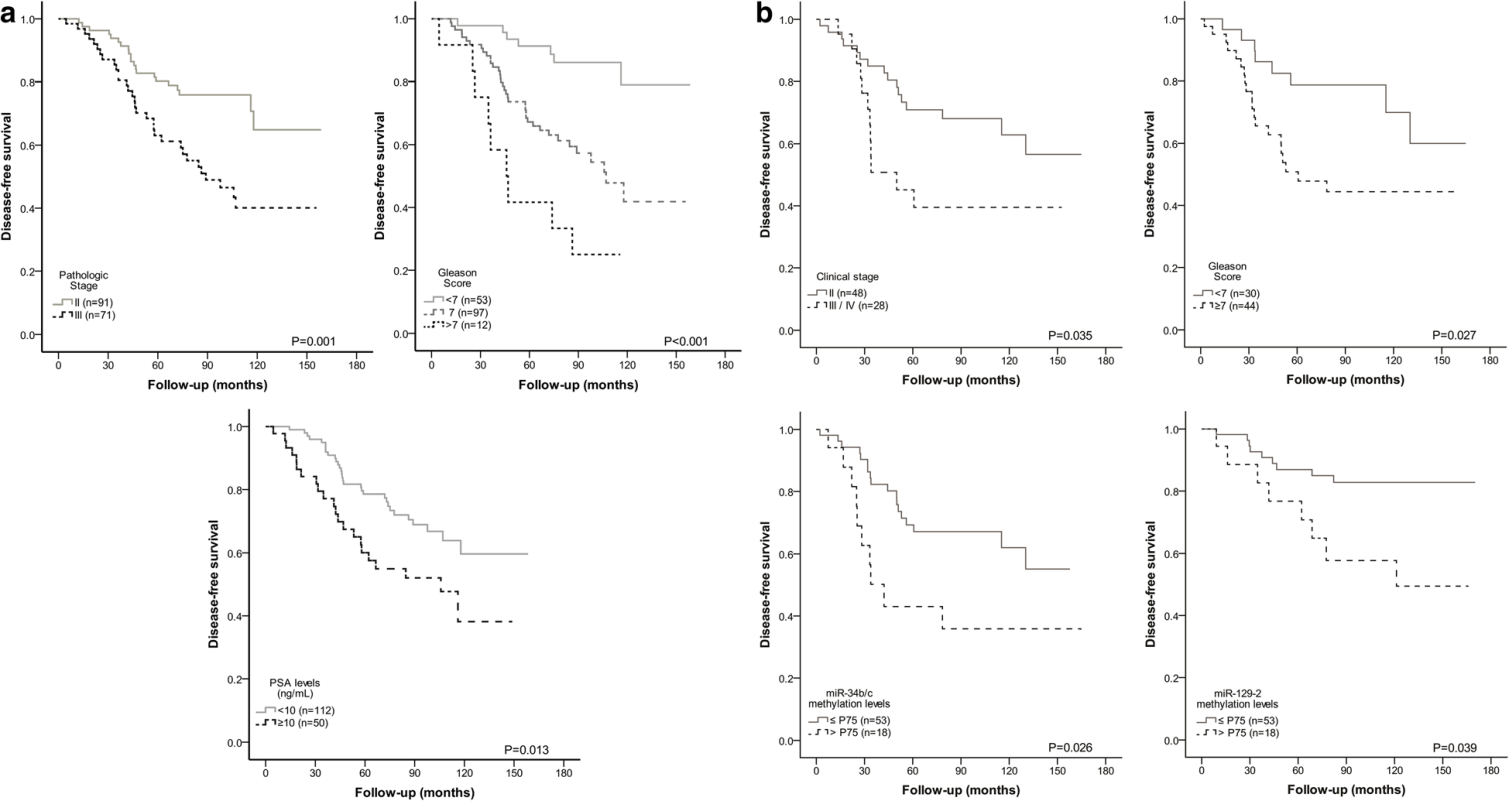
**a** - Disease-free survival (DFS) curves based on pathological stage (upper left panel) and Gleason score (upper right panel), and miR-129-2 methylation levels (lower panel) in Cohort #1. **b** - Disease-free survival (DFS) curves based on clinical stage (upper left panel) and Gleason score (upper right panel), miR-34b/c (lower left) and miR-129-2 (lower right) methylation levels in Cohort #2

**Fig. 6:**
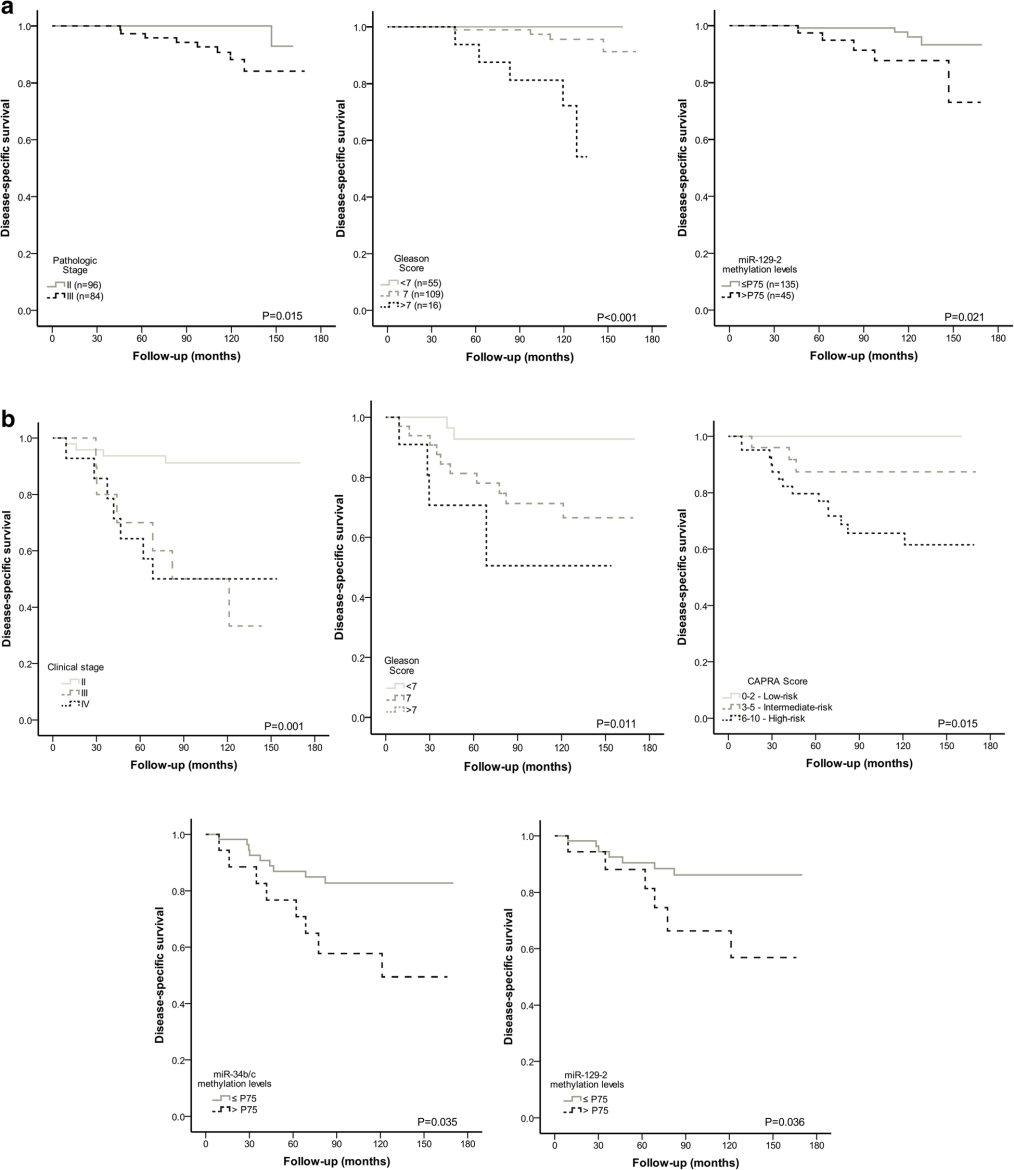
A - Disease-specific survival (DSS) curves based on pathological stage (upper left), Gleason Score (upper right), and miR-129-2 methylation levels (lower) in Cohort #1. B - Disease-specific survival (DSS) curves based on clinical stage (upper left), Gleason Score (upper center) CAPRA Score (upper left), miR-34b/c (lower left) and miR-129-2 (lower-right) methylation levels in Cohort #2

The median follow-up in Cohort #1 was 110.1 months (range: 2.8–169.1 months). Nine patients (5%) had died from PCa and 50 (28%) developed biochemical recurrence. Eighteen were never free of disease and were excluded from DFS analysis. In this cohort, pathological stage, GS and PSA levels significantly associated with DFS (Fig. 5A), whereas, only pathological stage and higher GS statistically associated with worse DSS (Fig. 6A). Remarkably, high miR-129-2 methylation levels associated with shorter DSS. In multivariable analysis, only GS and PSA levels for DFS and GS and miR-129-2 methylation for DSS retained prognostic value (Table 6).

**Table 6:** Cox regression analysis assessing the potential of clinical and epigenetic variables in the prediction of disease-specific survival and disease-free survival in the Cohort #1 and Cohort #2

Cohort #1							
Disease-specific survival – Cox regression analysis				Disease-free survival – Cox regression analysis			
Variable	HR	CI (95%)	*P*	Variable	HR	CI (95%)	*P*
Gleason Score				Gleason Score			
				<7	1		
≤7	1			7	3.96	1.76–8.91	0.001
>7	18.97	4.32–83.351	<0.001	>7	7.73	2.85–21.0	<0.001
miR–129-2				PSA			
≤ P75	1			<10	1		
> P75	6.12	1.56–24.07	0.009	≥10	1.87	1.07–3.26	0.027
Cohort #2							
Disease-specific survival – Cox regression analysis				Disease-free survival – Cox regression analysis			
Variable	HR	CI (95%)	*P*	Variable	HR	CI (95%)	*P*
Clinical stage				Clinical stage			
II	1			II	1		
III/IV	9.64	2.60-35.8	<0.001	III/IV	2.57	1.18-5.60	0.018
miR-34b/c				miR-34b/c			
≤ P75	1			≤ P75	1		
> P75	3.84	1.27–11.6	0.017	> P75	2.76	1.24–6.15	0.013

*HR* hazard ratio, *CI* confidence interval

Regarding Cohort #2, the median follow-up was 114.9 months (range: 10.3–170.1 months). Thirteen patients (17.6%) had died from PCa and 29 (39.2%) developed biochemical recurrence. In 3 patients, serum PSA levels >0.2 ng/ml persisted following treatment and these were not further considered for DFS analysis. Advanced clinical stage, higher GS, higher miR-34b/c and miR-129-2 promoter methylation levels statistically associated with worse DFS (Fig. 5B). In multivariable analysis only higher clinical stage and high miR-34b/c promoter methylation levels independently predicted shorter DFS (Table 6). Except for serum PSA, all clinicopathological parameters tested, as well as miR-34b/c and miR-129-2 promoter methylation levels associated with DSS in univariable analysis (Fig. 6B). Similarly to DFS, in multivariable analysis only clinical stage and high miR-34b/c promoter methylation levels independently predicted shorter DSS (Table 6).

## Discussion

PCa remains one of the most prevalent neoplasms and a leading cause of morbidity and mortality in men. Although PSA screening has decreased the number of men diagnosed with metastatic PCa, this was accomplished at the cost of overdiagnosis and overtreatment of a sizeable proportion of men carrying indolent/non-life threatening tumors [13]. Thus, a strong recommendation against serum PSA-based PCa screening has been issued [14], prompting the search for more effective biomarkers allowing for better risk stratification of PCa suspects. Herein, we aimed to tackle this clinical quest through discovery and preliminary validation of novel biomarkers for PCa detection and prognostication, using methylation analysis of microRNAs gene promoters.

Owing to our previous experience in DNA methylation analysis of PCa [6, 11], we searched for altered methylation patterns at the promoter regions of microRNAs deregulated in PCa. This information was then used to develop novel biomarkers, instead of microRNA expression levels, as previously attempted by other researchers [15]. Indeed, DNA methylation is easier to assess than microRNA expression, it is more specific and, importantly, more stable. Moreover, because microRNAs downregulation in cancer is more common than upregulation, it seemed likely that aberrant promoter methylation might constitute an underlying mechanism, similar to protein-coding genes [16]. Although several strategies might be used to identify microRNAs putatively downregulated due to promoter hypermethylation, high-throughput technologies such as methylation-array analysis are able to simultaneously pinpoint putative candidates [17] and the reliability of the results might be readily assessed through analysis of well-known hypermethylated loci. Indeed, results of the methylation array experiments confirmed the high prevalence of *GSTP1* and *APC* promoter methylation (data not shown), as we previously demonstrated in PCa [18]. To increase the likelihood of finding robust candidate biomarkers, we used stringent conditions based on high fold-variation of methylation levels between cancerous and non-cancerous tissue samples. From methylation-array analysis, six candidate microRNAs, putatively deregulated by promoter hypermethylation were identified. MiR-1258, miR-193b and miR-34b/c were the most promising candidates, displaying substantial PCa-specificity compared with other urinary tract tumors, an attractive feature for testing in bodily fluids. MiR-129-2 and miR-663a showed modest results and their inability to discriminate PCa from bladder cancer rendered it unsuitable for testing in urine samples.

Association between promoter methylation levels in tumor tissue samples and standard clinicopathological variables was also assessed. Higher miR-129-2 promoter methylation levels associated with higher GS and stage, suggesting prognostic value. MiR-34b/c, miR-663a and miR-1258 methylation levels also associated with pathological stage, but higher diagnostic performance underscores the potential for detecting PCa at early stages instead of prognostication, as we previously reported for *EFEMP1* promoter methylation [19]. Nevertheless, in this series of radical prostatectomies (Cohort #1) higher miR-129-2 methylation conveyed independent prognostic information, although only for DSS. Importantly, these results are in line with previous observations concerning the association of higher gene promoter methylation levels with clinicopathological features of more aggressive disease [11, 20].

Urine is a key sample to evaluate DNA methylation biomarkers for PCa, as it is readily collected and biomarkers are diluted to a smaller extent than in plasma, providing higher sensitivity[21]. Nevertheless, the amount of DNA potentially deriving from prostatic cells is variable, usually low, entailing the use of a panel with limited number of biomarkers. Thus, only miR-34b/c, miR-193b and mir-1258, were tested in urine samples (Cohort #3). From these, Mir-193b was previously shown to be aberrantly methylated in PCa cell lines as well as in primary tumors, but no data is available regarding its performance as PCa detection biomarker [22, 23]. Indeed, Mir-193b performed best, with high AUC, sensitivity, specificity and PPV, whereas miR-34b/c performance was more modest.

Intriguingly, miR-1258, which showed the best performance in tissue samples (Cohort #1), displayed a strikingly different result in urines as its methylation levels were higher in controls than in PCa patients. The reason for this discrepant result is not immediately apparent, but it might be due to high miR-1258 promoter methylation in non-epithelial cells, such as leucocytes, which are relatively more abundant in urine than in tumor tissue samples. Moreover, median miR-1258 promoter methylation levels in urines from PCa were substantially inferior to those of miR-193b, impairing the robustness of the assay. It should be recalled that, contrarily to other studies, the urine samples we used were not collected following DRE or prostatic massage, which are usually employed in an attempt to increase sensitivity. Studies dealing with PCa biomarkers in urine vary in the method of urine collection and the real impact of prostatic massage has never been evaluated [24]. It could be argued that the distance from the peripheral zone to the urinary tract flow may render urinary based tests less sensitive, which would be an important issue since most malignancies arise from this zone. Nevertheless, studies on *PCA3* did not find a difference in the levels of this biomarker between patients with peripheral versus transitional zone PCa [25, 26].

Currently, the performance of serum PSA and urinary *PCA3,* the only biomarkers approved for clinical use is rather limited. The reported performance of serum PSA as PCa biomarker is somewhat modest, with AUC ranging from 0.54 to 0.70 [27, 28]. Even other serum PSA-derived measurements, like PSA-density, free PSA percentage and PSA-velocity have not significantly improved performance [28]. Nonetheless, *PCA3*, which was reported to perform better than serum PSA both in urine and ejaculates but has not been approved for population-based screening, displays AUCs varying from 0.66 to 0.79 [27–30]. Additionally, although miRs’ expression has been extensively investigated in liquid biopsies, available data for urine samples is rather limited. Nevertheless, an AUC of 0.74 was reported for miR-107 [31] and simultaneous quantification of miR-107 and miR-574-3p in urine showed an AUC of 0.83, for PCa cancer detection [32]. We should emphasize that in our dataset, urinary miR-193b promoter methylation (AUC = 0.96) outperformed not the only currently approved clinical biomarkers, but also the previously mentioned miRs, constituting a promising tool for non-invasive PCa detection.

Because a major goal of this study was to discriminate clinically aggressive from indolent PCa, it was critical to test the prognostic value of microRNAs in a pre-therapeutic setting, which was accomplished in series of prospectively collected prostate biopsies (Cohort #3). In univariable analysis, most standard clinicopathological parameters associated with DFS and DSS, clinically validating this dataset. The same was demonstrated for higher miR-129-2 and miR-34b/c promoter methylation levels. The CAPRA score, however, only associated with DSS but not DFS. This was unexpected as its determination at diagnosis associated with DFS in patients with clinically localized disease submitted to RP [12]. Notwithstanding, our prostate biopsy series included PCa at diverse clinical stages, submitted to different therapeutic modalities (RP, radiotherapy, androgen-deprivation therapy), which might explain the apparent flaw of CAPRA score. In multivariable analysis, only clinical stage, amongst all clinicopathological parameters, retained independent prognostic value, both for DFS and DSS. Remarkably, high miR-34b/c promoter methylation levels also predicted shorter DFS and DSS, suggesting that it might constitute a useful PCa prognostic biomarker. These results suggest that high miR-34b/c promoter methylation levels identify clinically aggressive PCa, irrespective of disease extent at diagnosis.

It should be acknowledged that in spite of the excellent diagnostic performance of miR-193b promoter methylation in urine, additional patient sets must be tested. Furthermore, a larger cohort of patients submitted to biopsy and subjected to different therapies is required to further validate our observations. Ultimately, we plan to develop a multiplex assay to simultaneously assess miR-193b, miR-129-2 and miR34b/c promoter methylation, allowing for diagnostic and prognostic assessment of PCa suspects in a single analysis.

## Conclusion

Through genome-wide screening, a set of methylation-based PCa biomarkers was identified and validated. MiR-193b demonstrated high sensitivity and specificity for detection of PCa, both in tissue and urine, whereas high miR-129-2 and miR-34b/c methylation levels independently predicted for shorter DSS and DFS or DSS, respectively. If confirmed in larger and independent datasets, quantitative promoter methylation of selected miRs might provide useful tools for clinical management of PCa patients. The authors would like to extend their appreciation to all the patients and control subjects that kindly provided the biological samples for this study.

## Additional files

Additional file 1: Table S1. Primer sequences used for reference gene and each microRNA promoter methylation analysis. (DOC 28 kb)
Additional file 2: Figure S1. Distribution of miR’s promoter methylation levels in prostatic, vesical and renal tissues. MNPT - morphologically normal prostatic tissue; PCa - prostate cancer; NBl - normal bladder; BlCa - bladder cancer; NK - normal kidney; RCT – renal cell tumor. (TIF 1338 kb)

## Abbreviations

ACTB: β-actin
ADT: Androgen deprivation therapy
APC: Adenomatous polyposis coli
AUC: Area under the curve
BlCa: Bladder cancer
CAPRA: Cancer of the Prostate Risk Assessment
CI: Confidence interval
DFS: Disease-free survival
DNA: Deoxyribonucleic acid
DSS: Disease-specific survival
EFEMP1: EGF-containing fibulin-like extracellular matrix protein 1
GS: Gleason score
GSTP1: Glutathione S-transferase P
HD: Healthy donnors
HR: Hazard ratio
MNPT: Morphologically normal prostate tissue
NBl: Normal bladder
NK: Normal kidney
NPV: negative predictive value
PBS: Phosphate-buffered saline
PCa: Prostate cancer
PCA3: Prostate cancer antigen 3
PPV: Positive predictive value
PSA: Prostate-specific antigen
RCT: Renal cell tumor
RNA: Ribonucleic acid
ROC: Receiver operator characteristics
RP: Radical prostatectomy
