# Supplementary material for: MiR-193b promoter methylation accurately detects prostate cancer in urine sediments and miR-34b/c or miR-129-2 promoter methylation define subsets of clinically aggressive tumors
Source: Mol Cancer. 2017 Jan 31;16:26. doi: 10.1186/s12943-017-0604-0 (PMC5282784; doi:10.1186/s12943-017-0604-0)
Supplement: Additional file 1: Table S1. — Primer sequences used for reference gene and each microRNA promoter methylation analysis. (DOC 28 kb) [file 12943_2017_604_MOESM1_ESM.doc]

**Sup Table 1** - Primer sequences used for reference gene and each microRNA promoter methylation analysis.

| **Gene** | **Forward (F)** | **Reverse (R)** |
| --- | --- | --- |
| ***ACTB*** | TGGTGATGGAGGAGGTTTAGTAAGT | AACCAATAAAACCTACTCCTCCCTTAA |
| **miR-34b/c** | GTTTAGTTACGCGTGTTGTGC | CGAAAAACGCCCTACCATA |
| **miR-129-2** | CGGCGAATCGAAGAAGTC | TACGCCCTCCGCAAATAC |
| **miR-152** | TCGTCGTTCGGGATTTTC | ACTAACCACGTCCGCACC |
| **miR-193b** | AGCGGGGTGTTTGTGTTC | AAACATAAACGACGCATTCCA |
| **miR-663a** | GGGATAGCGAGGTTAGGTC | CATTCGTAACGAATAAAACCC |
| **miR-1258** | TCGGTATATTTGGCGGAGTC | TCCGACGAAAATAAACCCC |
