# Supplementary figures and images for: MiR-193b promoter methylation accurately detects prostate cancer in urine sediments and miR-34b/c or miR-129-2 promoter methylation define subsets of clinically aggressive tumors
Source: Mol Cancer. 2017 Jan 31;16:26. doi: 10.1186/s12943-017-0604-0 (PMC5282784; doi:10.1186/s12943-017-0604-0)

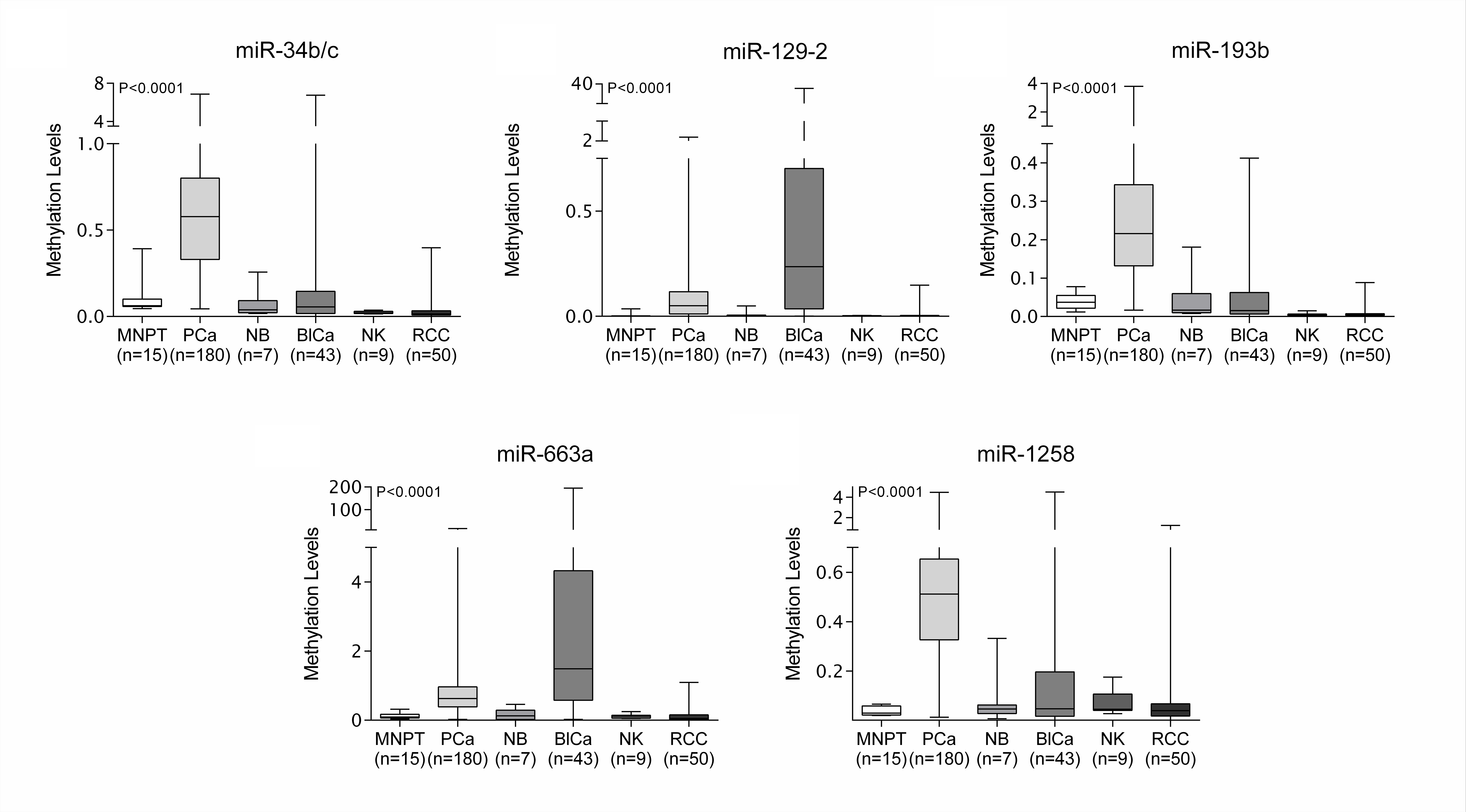

Supplement: Additional file 2: Figure S1. — Distribution of miR’s promoter methylation levels in prostatic, vesical and renal tissues. MNPT - morphologically normal prostatic tissue; PCa - prostate cancer; NBl - normal bladder; BlCa - bladder cancer; NK - normal kidney; RCT – renal cell tumor. (TIF 1338 kb) [file 12943_2017_604_MOESM2_ESM.tif]
